# European Correspondence

**Published:** 1877-09

**Authors:** R. J. Nunn

**Affiliations:** Manchester, England


MANCHESTER, ENGLAND, August 11, 1877.

A week spent at the meeting of the British Medical Association has been most instructive; not that the discussions were any more scientific, the subjects more lucidly explained, the conclusions more logical, or the discoveries more novel, than would be found at our own association.

England teaches from our text-books, uses our instruments, adopts our treatment, praises our doctors, but says they shall not practice within her borders. Free trade is her motto, but she does not apply the principle to physic. Yet, when within a year her neighbor, France proposed to adopt the English maxim, and allow only French doctors to practice in France, England was the first to make an outcry, and the matter was even deemed of so much importance as to be brought before her parliament. Surely this is a question which might well be taken up by the International Medical Congress, to the end that some uniformity of education, and of professional recognition might be brought about, or at least placed within the reach of the medical men of each country, without entailing on them the necessity of traveling over the world, and spending a lifetime and a fortune, preparing for and passing examinations, according to the individual requirements of each school by which they desire recognition.

The address on medicine, of Wm. Roberts, M.D., F.R.S., on "The Doctrine of the Contagium Vivum and its Applications to Medicine," was very practical, and a resume will not be uninteresting.

The speaker pointed out the close resemblance between a contagious fever and the fermentation produced by yeast. He also showed another form of fermentation produced in hay infusion inoculated with "bacillus subtilis," a bacterium very common in vegetable infusions, and in curdling milk. This bacillus subtilis and the yeast plant may be taken as representatives of a class of organisms which are grouped together under the general designation of *saprophytes*, which is intended to include under one head all the organisms connected with the decomposition and decay of organic matter The first proposition he set himself to prove was "that organic matter has no inherent power of generating bacteria, and no inherent power of passing into decomposition;" the second, "that bacteria are the actual agents of decomposition;" the third, "that the organisms, which appear as if spontaneously in decomposing fluids, owe their origin exclusively to parent germs derived from surrounding media."

To support these propositions he brought forward three sets of preparations of organic mixtures and liquids, all of them in bottles, the necks of which were closed by plugs of cotton wool, which protected the contents from the dust of the air, but left it freely open to its gaseous elements.

The first set had been rendered sterile, by the prolonged application of the heat of boiling water. The second had been filtered through unglazed earthenware. The third set consisted of fluids, such as blood, urine, pus, etc., which had been simply removed from the interior of the living body, and transferred, with proper precautions, with the glass vessels previously purified. All these had been in the possession of the speaker for months, many of them for years. They had all been exposed to conditions the most favorable for decomposition, but none of them showed the least change.

Much of the confusion which has hitherto surrounded this subject has arisen from ignorance of the high degree of heat which the spores of these organisms could endure without having their vitality destroyed. For example, it has been found that while the living "septic monads" are killed by a heat of 140 degrees, Fahrenheit, the spores of one variety, which are so minute as to be invisible to the highest microscopic powers, except when they exist in masses, are capable of germinating after being subjected to a heat of 300 degrees, Fahrenheit, for ten minutes; and here the speaker made a truly pertinent remark, to the effect that the abioginist must not look among the saprophytes in hopes of finding an example of spontaneous generation. Looking at the order of life as it now exists on the earth, it may be described as beginning with a chlorophyll body, and ending with a saprophyte. The office of the chlorophyll body being to form organic compounds from inorganic matter, while the action of saprophyte is one of progressive disintegration, and finally a breaking up of all the organic compounds it contains into carbonic acid and ammonia.

The main object of this address appears to be to assert the truth of the doctrine of a contagium vivum, and the coclusion that that contagium consists of an independent organism or parasite. As examples to sustain this position, three diseases were selected, viz: septicæmia, relapsing fever, and splenic fever; and it was shown that septic bacteria are the cause of septicæmia, sporilla the cause of relapsing fever, and the bacillus anthracis the cause of splenic fever. Yet this bacillus anthracis is identical in appearance with the bacillus subtilis seen as a ferment in the hay infusion, the one a harmless saprophyte, the other a deadly contagium. This change might be the result of the laws of variation known to exist in the animal and vegetable kingdoms, and which apply in a curiously exact manner to many of the phenomena of contagious diseases. Parallel instances may be found in the bitter almond as a sport from the sweet almond, the nectarine from the peach, diphtheria from scarlet fever, etc. Again, the history of cholera epidemics looks as if the cholera virus were an occasional sport from some Indian saprophyte, which, by variation, has acquired a parasitic habit, and having run through countless generations, either dies out or reverts to its original type; and so with typhoid fever as a sport from some saprophyte common to our sewers.

Pathophytes, as these pathogenic organisms have been termed, are, as far as our present knowledge extends, all to be found in that group of fungi called *bacteria.* Now, fungi present a marked tendency to take on a parasitic habit, and some of them have a special fermentive action. In some diseases, as septicæmia for example, both these processes may play a part, the ferment pervading and deorganizing the system, and then the organisms feed upon the dead or decaying tissues.

Such is the gist of this very able discourse, but it would be going too far to say that this settled the question in favor of "contagium vivum;" doubtless among the audience were many who, while appreciating the marked ability of the address, differed "in toto" from the deductions. It is to be regretted that no discussion of the subject took place, as the record of the opinion of the many observers assembled on that occasion would have been most interesting and instructive.

R. J. Nunn, M.D.

